# Mr. Ward, on Hydrophobia

**Published:** 1807-05

**Authors:** M. Ward

**Affiliations:** Manchester


### *To the Editors of the Medical and Physical Journal.*

Gentlemen,

In my remarks on the case of William Miles, inserted in your last Number, I omitted to notice a few passages in Mr. Hicks's narrative, which appear to me to afford additional evidence in favour of the arguments I had occasion to advance.

In page 272 we are told, that the patient appeared much agitated, and (as his mother says) "his throat projected almost to his chin;* *he grated his teeth very much, and appeared incapable of swallowing his saliva, which he continually attempted to do.*"

Upon each of these observations I beg leave to offer a few Remarks.

The first of these, namely, the extraordinary projection or swelling of the throat, exceeds every thing of this kind, as far as I know, in the history of any disease that has been hitherto described, except it be bronchocele, obesity, or cynanche parotidea.† Even in *hysteria*, during the continuance

---

* This was on Sunday, Nov. 30, and it appears from the following passage, that Mr. Hicks examined his throat on Monday, Dec. the 1st. when the swelling still continued.

"I then asked Miles how he felt himself; he said, he felt pain in the part of his leg the dog had bit, which extended up the inner side of his thigh, to the pit of his stomach, and which felt to him as tight as if a strong cord was bound round him, from that part, up to his throat, *(which appeared swollen)* with great difficulty in swallowing."

† In the only case of this disease that has fallen under my care, not only the parotids, but all the maxillary and other glands in the neighbourhood of tinuance of the most violent paroxysms, I have never been able to detect more than a slight protuberance of that part of the throat, which is the usual seat of the globus hystericus; and in hydrophobia, I recollect at present only one instance, (which is that related by Dr. Munckley in the 2d volume of the Medical Transactions) where any external appearance of enlargement is said to have taken place; though I would by no means be understood to infer, that a slight degree of fulness about the upper part of the trachea may not have happened in other cases.

2d. The grating of the teeth will admit of a very easy and satisfactory explanation, upon the principles which were offered in my last paper; it being well known, that nothing is more common in trismus,* whether arising in hysteria or tetanus, than for the teeth to be pressed or "grated" together, upon every increase of the spasms.— 3d. Nor, I believe, is any thing more *rare* in hydrophobia, than for the patient to be "*continually attempting to swallow his saliva*;" on the contrary, I have always understood it was to avoid the anguish occasioned by every attempt of this kind, (or rather to avoid being suffocated) that he labours so incessantly to get rid of it, by spitting and throwing it from him with great vehemence, and in every possible direction; a symptom from which Mr. Hicks's patient appears, from no notice being taken of it, to have been entirely free; which is very remarkable, because, as he appears from his mother's account, to have been "incapable of swallowing his saliva," it might have been expected, (supposing the disorder to have been hydrophobic) that the usual consequences of the ineffectual attempts to swallow, would have ensued; such as spasms in the muscles of deglutition, &c. difficulty of breathing, a frequent and copious discharge of the saliva, &c.; circumstances which are not once mentioned as having taken place in the whole course of the disease;† though, whenever they

 do

---

of the throat and fauces, were swelled to such an enormous size, as gradually to impede, and at length put a stop to the respiration.

* The grating, or grinding of the teeth, the propensity to bite, and the continual attempts to swallow the saliva, are convincing proofs in my mind of a pretty strong tendency at those times to a locked jaw, which might probably be prevented from being fully formed by the want of power in the proximate cause, and by the mobility of the sensorial power being such as to prevent the spasms from continuing long together in one particular set of muscles.

† Mr. Hicks frequently speaks of "the spasmodic affection of the præcordia"

do occur, (particularly the latter) they occasion so much distress to the patient, and inconvenience to the attendants, that it is impossible for them to escape being observed.

His manner of expressing his dislike to the sight of water too, by "*throwing his head violently back, and saying it terrified him,*" bears a much nearer resemblance (if it can be said to resemble any thing that has before occurred) to the spasms which take place in opisthotonos, than to the gasping for breath, accompanied with an inability of utterance,* the frightful contortions of the muscles of the face, chest and limbs, which the sight of water very rarely fails to produce in hydrophobia.

It also strengthens the opinion I have endeavoured to maintain relative to the nature of the disease, to observe, that though the patient is said to have "swallowed with difficulty, more so at some times than at others, as the spasms were more or less violent," yet it does not appear that he was unable to swallow liquids at any time during his confinement; on the contrary, it is reasonable to infer, both from the general tenor of the history, and from the large doses of the medicine, which were given every two hours, between the time when Mr. H. was first called on the Sunday evening, till the time of the patient's sudden amendment on the Saturday following; that the difficulty, in whatever degree it might exist, was not insurmountable.† The case, however, is curious and interesting, as it tends

---

cordia and fauces, and the tightness of his stomach and throat, being increased *upon pressure being applied to the cicatrix.*" He also says, (see page 274, 5) "I called on him again on Friday evening, Dec. 5, and learned from those who attended him, that the spasmodic affection of his throat, &c. became much increased on the preceding day, (Thursday) so much so, that he appeared greatly agitated at the sight of water, and would not suffer it to approach his lips, *throwing his head violently back when it was brought to him, saying it terrified him.* He expressed the same aversion to water on Friday evening, at the time I was with him, and which had continued from the Thursday. On the Sunday, Monday, Tuesd[illegible]y, and Wednesday, *he did not appear to have a greater aversion to water than to other fluids.* From the first he swallowed fluids with difficulty, more so at some times than at others, as the spasms were more or less violent."

* Though in many instances the hydrophobic patient has recourse to the most earnest intreaties, or if these fail to induce his persecutor to desist from his ill timed offers of liquids, &c. to the most vehement remonstrances.

† I find, on re-examining Mr. H's account of W. M's case, I have stated the time which intervened between Sunday evening (Nov. 30) and the Saturday morning following (Dec. 6) at seven days instead of five days and a half; tends to confirm the analogy which subsists between tetanus and hydrophobia; it also furnishes another instance, in addition to the many which are upon record,† of the influence of the imagination upon the human body; it likewise tends to illustrate, as well as to corroborate, some of the principles which I took the liberty to propose in the 11th vol. of the Med. and Phys. Journal, p. 540, &c.; and also in a P. S. to my last communication, inserted in No. 98, of the same work: but these are points which I shall not attempt to enlarge upon at present.

I am, &c.

*Manchester, April* 12, 1807. M. WARD.

a half; a mistake which might easily occur from the haste with which my remarks were composed, and which I am glad to have this opportunity to rectify; though it does not at all affect the object for which the calculation was made, which was to show the quantity of liquid medicine taken in a given time, which was the same in both cases, and which was such as to be totally incompatible with the idea of hydrophobia. The aggregate amount of the different articles, *taken in five days and a half*, will then stand thus: Tinct. of opium, four ounces and a drachm; compound spirit of ammonia, eight ounces and a quarter; tinct. of assafœtida, 16 ounces and a half; camphorated mixture, one hundred and twenty-one ounces and a half; or upwards of seven pints and a half, ale measure.

† A case extremely similar to the present in all the leading circumstances, (as well as in the event) is related by Dr. Shadwell in the 3d vol. of the Mem. of the Med. Soc. of Lond. p. 464; and another by Dr. Wrightson, vid. Med. Trans. But in these, and many other instances, where the symptoms appeared so early as ten or twelve days after the bite, (or where no bite has been received) the disease may with much more probability (as far as it resembles hydrophobia) be attributed to terror operating upon the mind, than to rabid infection; in which light they are, I believe, generally considered. See Hamilton on Hydrophobia, vol. i. p. 254, &c.

